# Predicting hemorrhage expansion in patients with hypertensive intracerebral hemorrhage: the HE-VSD-A2TP score

**DOI:** 10.3389/fneur.2025.1634441

**Published:** 2025-07-01

**Authors:** Yingying Zhu, Luyang Lin, Wei Wang, Conghui Liu, Peiling Dai, Kai Chen, Jian Chen

**Affiliations:** ^1^Department of Emergency Medicine, The Second Affiliated Hospital and Yuying Children's Hospital of Wenzhou Medical University, Wenzhou, Zhejiang, China; ^2^Department of Ultrasound Imaging, The First Affiliated Hospital of Wenzhou Medical University, Wenzhou, Zhejiang, China; ^3^Department of Radiotherapy, The First Affiliated Hospital of Wenzhou Medical University, Wenzhou, Zhejiang, China; ^4^Department of Cardiovascular and Thoracic Surgery, The Second Affiliated Hospital and Yuying Children's Hospital of Wenzhou Medical University, Wenzhou, Zhejiang, China

**Keywords:** intracerebral hemorrhage, hematoma expansion, predictive model, risk stratification, clinical decision-making

## Abstract

**Background:**

Hematoma expansion (HE) in hypertensive intracerebral hemorrhage (HICH) is significantly associated with patient mortality. Early identification of HE would be planning for appropriate and aggressive management for improving outcome and containing HE. Existing HE prediction models show variable accuracy across settings. To address this limitation, we developed and validate a new predictive model to enhance the accuracy of HE in patients with HICH.

**Methods:**

We conducted a retrospective cohort study using data from two centers. The primary outcome was the occurrence of HE within 24 h of symptom onset, defined as an increase in hematoma volume ≥33% or ≥12.5 mL on follow-up imaging. Logistic regression was used to identify independent predictors of HE, and the HE-VSD-A_2_TP score system was developed and validated.

**Results:**

Five hundred and sixty seven patients in the derivation cohort and 378 patients in the validation cohort. The HE-VSD-A_2_TP score included age, uncontrolled blood pressure, hematoma volume, irregularity/lobulation shape, non-homogeneous density, presentation within 6 h from symptom onset to CT, and the use of anticoagulation/antiplatelet therapy. The HE-VSD-A2TP score demonstrated superior discrimination in predicting HE compared to existing models like PREDICT, 9-point, and BRAIN scores, with an AUC of 0.871(95%CI 0.839–0.904) in the derivation cohort and 0.858 (95%CI 0.819–0.897) in the validation cohort. The score also showed excellent calibration and outperformed other models in terms of sensitivity, specificity, likelihood ratios, negative predictive value, and positive predictive value. With regard to clinical usefulness, the decision curve analysis (DCA) of HE-VSD-A_2_TP showed higher net benefit than PREDICT, 9-point, and BRAIN scores in the both cohorts.

**Conclusion:**

The HE-VSD-A2TP score was validated to be an effective tool for identifying patients at risk of HE in patients with HICH. It was a valuable tool for guiding clinical management strategies and potentially improving patient outcomes.

## Introduction

Hypertensive intracerebral hemorrhage (ICH), a catastrophic stroke subtype, is associated with high mortality and morbidity rates (1). Hematoma expansion (HE), which occurs in a substantial proportion of ICH patients, serves as a crucial predictor of poor outcomes (2). Identifying patients at high risk of HE is essential for clinical management strategies, including surgical intervention, and ultimately influences patient prognosis (3), which has driven the development of various predictive models (4–7).

Despite the advances in neuroimaging and medical technologies, the prediction of HE remains a clinical challenge. The BRAIN score (5), PREDICT score (6), and the 9-point clinical prediction score (7) are some of the established models for predicting HE. These models incorporate variables such as baseline hematoma volume, time from symptom onset to baseline CT, use of anticoagulants, intraventricular hemorrhage, and admission Glasgow Coma Scale (GCS) (5–7). However, these scoring systems have varying predictive abilities in different environments and their overall performance is not good (8). While established models like the PREDICT Score and the 9-point clinical prediction model incorporate the CTA spot sign, they may not be universally applicable due to the limited availability of advanced imaging in certain settings (9). Moreover, models focusing solely on baseline hematoma volume or initial GCS fail to incorporate the dynamic changes in patient conditions and the multitude of biological factors contributing to HE. As research continues to deepen, many new and meaningful factors for HE have not been incorporated into the existing prediction systems (10).

Given the limited generalizability in existing models, we aimed to develop a universally applicable score integrating novel non-contrast CT (NCCT) markers and dynamic clinical factors to improve HE prediction in ICH. The new model seeks to provide a reliable alternative for settings where a CTA is not feasible. Additionally, while CTA is valuable for identifying underlying vascular anomalies, the focus here is on enhancing predictive accuracy specifically for HE in cases confirmed as hypertensive ICH through clinical assessment and available imaging. This approach aims to improve prognostication and guide clinical management in resource-constrained environments.

## Methods

### Study design and participants

We conducted a retrospective cohort study utilizing data from two centers: The Second Affiliated Hospital of Wenzhou Medical University (Derivation cohort) and The First Affiliated Hospital of Wenzhou Medical University (Validation cohort). The study period for the derivation cohort spanned from 1 June 2018 to 28 February 2024, while the validation cohort data was collected from 1 August 2021 to 28 February 2024. Participants were patients aged ≥18 years with hypertensive ICH (HICH). HICH was defined by the presence of ICH with a history of hypertension. Exclusion criteria included: patients who underwent surgical treatment before the repeat CT scan; hemorrhage secondary to cerebral arteriovenous malformations, trauma, intracranial aneurysms, brain tumors, or hemorrhagic transformation of brain infarction; coagulopathy-related brain hemorrhage, and no repeat head CT scan within 24 h after the initial CT scan. This study was approved by the Ethics Committee of the Second Affiliated Hospital of Wenzhou Medical University. The informed consent was waived due to the retrospective nature.

### Patient identification

We identified patients through a comprehensive electronic medical records system at both centers. The search criteria included relevant International Classification of Diseases (ICD) codes for HICH, as well as key terms associated with HICH. The medical records of potential participants were manually reviewed by two researchers to ensure that all inclusion and exclusion criteria were met.

### Sample size

To ensure the robustness of our predictive model, we adhered to the principle of Events Per Variable (EPV), which is a widely accepted criterion for determining the adequate sample size in logistic regression analysis (11). The EPV principle recommends that there should be at least 10 events (or cases) per predictor variable to avoid overfitting and to ensure the stability of the regression coefficients. Considering an expected event rate of approximately 20–30% and allowing for a dropout rate of 10%, we estimated that we would need a total sample size of at least 500 patients in the modeling cohort to observe approximately 100 events. For the validation cohort, we aimed to include at least 200 patients to validate the model derived from the modeling cohort, ensuring that the model’s predictive performance could be generalized to an independent set of patients.

### Variables

Candidate variables included demographics, medical history, clinical presentation, laboratory results, and imaging findings. Specific variables were selected based on their potential association with HE, as informed by previous literature and clinical expertise. Demographic (Age, Gender), vital signs upon Emergency Department (ED) arrival, GCS, anticoagulant/antiplatelet therapy, time from symptom onset to head CT scan, baseline hematoma volume (calculated using the ABC/2 method or volumetric analysis), hematoma shape (irregularity, lobulation), and non-homogeneous density which was manifesting as any of the following signs: blend sign, hypodensities, black hole sign, swirl sign, island sign, or liquification, biochemical indicators (white blood cell count, hemoglobin, platelet count, serum glucose, serum creatinine, international normalized ratio (INR)). Hypodensities, defined as areas of lower attenuation; liquification: presence of fluid levels within the hematoma; Island sign was defined as ≥3 separate small hematomas dispersed and distinct from the main hematoma; or ≥4 small hematomas, several of which can be connected to the main hematoma. Black hole sign was characterized as a well-demarcated hypodense area within the hyperdense hematoma, which may be round, oval, or strip-shaped, and does not connect with the surrounding brain tissue, with a CT value difference of at least 28 Hounsfield Unit (HU) from the surrounding hematoma. Swirl sign was described as a hypodense or isodense area within the hyperdense hematoma, with variable shapes such as round, strip-like, or irregular. Blend sign: Refers to the coexistence of relatively hyperdense and hypodense areas within the hematoma, with a clear boundary between them, and a CT value difference of more than 18 HU (10). Uncontrolled blood pressure was defined as systolic blood pressure (SBP) ≥ 140 mmHg recorded within 1 h of hospital admission.

### Outcome

The primary outcome was the occurrence of HE within 24 h of the onset of symptoms, defined as an increase in hematoma volume ≥33% or ≥12.5 mL on follow-up imaging (5).

### Statistical analysis

Continuous variables were presented as mean ± standard deviation (SD) or median with interquartile range (IQR), depending on the distribution. To compare baseline characteristics between two cohorts, the student’s *t*-test or Mann–Whitney *U* test was used for continuous variables, as appropriate. For categorical variables, the chi-square test or Fisher’s exact test was applied.

For the derivation of the predictive model for HE in patients with HICH, we employed a rigorous statistical approach. Candidate variables were selected based on two criteria: (1) their established link to HE as reported in existing literature, and (2) their availability in routine hospital settings without requiring additional intervention for assessment. This variable selection ensured that all variables included in the final model would be clinically relevant. We utilized a standard logistic regression model with HE (yes/no) as the dependent variable. All preselected variables were simultaneously entered into the model. Variables were retained in the final model only those with *p* value<0.05 from the regression analysis. We aimed to construct a predictive scoring system based on the final model. The scoring system was developed by dividing the regression coefficient of each predictor variable by the smallest coefficient from the model, thereby assigning a whole number or half-point score to each variable.

The new score’s discrimination for HE was examined by the area under the receiver operating characteristic curve (AUC) and calibration was conducted using calibration curves and the Hosmer and Lemeshow (H–L) test. The DeLong’s non-parametric method was used to compare the AUC values. To assess the clinical utility of the new score in predicting HE, Decision Curve Analysis (DCA) was conducted to compare the net benefits of the new score, BRAIN, PREDICT and the 9-point across various threshold probabilities.

Missing data were predominantly observed in laboratory indicators, with the rate of missingness being less 5% for each indicator. Missing data filling is carried out by using the multiple imputation. Since our prediction model is not intended to incorporate laboratory indicators, missing data has no impact on our model construction. All analyses were conducted using R, version 4.4.0 (R Foundation for Statistical Computing, Vienna, Austria), and a *p* value < 0.05 was considered to be statistically significant.

## Results

### Populations

**Figure 1:**
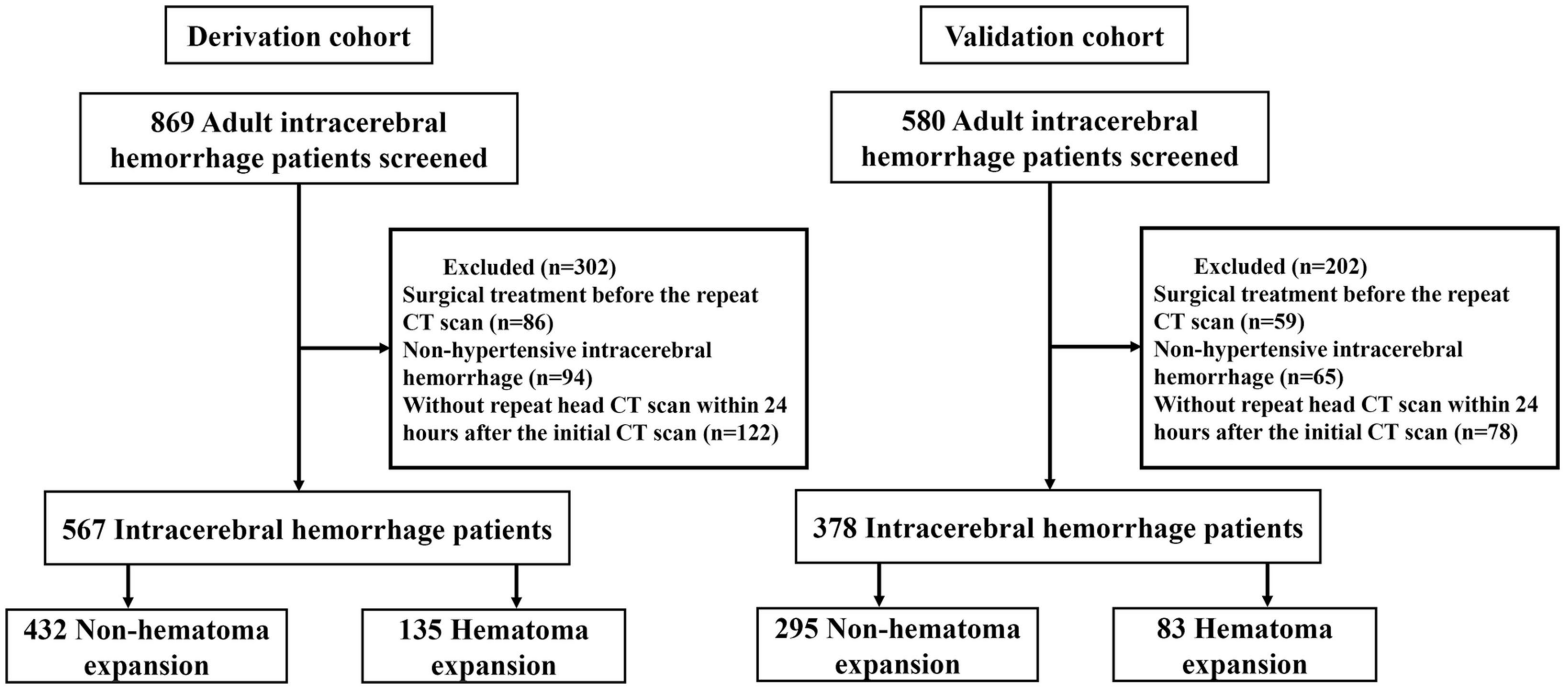
The study flow diagram.

In our analysis, the derivation cohort encompassed 567 patients, and the validation cohort comprised 378 patients (Figure 1). While the baseline characteristics of the two cohorts were largely similar, there were notable differences in vital signs upon admission and imaging features, specifically hematoma volume and density characteristics. Despite these differences, the incidence of HE was comparable, occurring in 23.8% of the derivation cohort and 22% of the validation cohort. A detailed comparison of the baseline characteristics of both cohorts is presented in Table 1.

**Table 1:** Baseline patient characteristics in derivation and validation cohort.

Variables	Derivation (*N* = 567)	Validation (*N* = 378)	*P*-value
Demographics			
Age (years), Median (IQR)	68 (60–75)	65 (62–70)	0.078
Male sex, *n* (%)	325 (57.3)	196 (51.9)	0.112
Comorbidities			
Diabetes mellitus, *n* (%)	102 (18.0)	60 (15.9)	0.449
Hypertensive heart disease, *n* (%)	54 (9.52)	42 (11.1)	0.496
Chronic kidney disease, *n* (%)	32 (5.64)	18 (4.76)	0.656
Atrial fibrillation, *n* (%)	28 (4.94)	15 (3.97)	0.588
Prior intracerebral hemorrhage, *n* (%)	11 (1.94)	5 (1.32)	0.643
Prior cerebral infarction, *n* (%)	12 (2.12)	4 (1.06)	0.328
Vital signs on admission			
T (°C), Median (IQR)	37.0 (36.8–37.3)	36.9 (36.7–37.2)	0.002
RR (breaths/min), Median (IQR)	24 (22–26)	23 (21–26)	0.016
SBP (mmHg), Median (IQR)	173 (161–184)	171 (160–182)	0.345
DBP (mmHg), Median (IQR)	98 (93–102)	94 (89–100)	<0.001
HR (bpm), Median (IQR)	109 (103–115)	110 (106–115)	0.001
GCS, point	14 (13–15)	14 (13–14)	0.007
Imaging characteristics			
Hematoma volume, *n* (%)			<0.001
≤10 mL	244 (43.0)	211 (55.8)	
>10- < 20 mL	197 (34.7)	113 (29.9)	
≥20 ml	126 (22.2)	54 (14.3)	
Hematoma shape, *n* (%)			
Irregularity/lobulation	200 (35.3)	122 (32.3)	0.377
Hematoma density, *n* (%)			
Non-homogeneous density†, *n* (%)	246 (43.4)	194 (51.3)	0.020
Other characteristics			
Anticoagulation/antiplatelet, *n* (%)	75 (13.2)	66 (17.5)	0.090
Uncontrolled blood pressure‡, *n* (%)	277 (48.9)	191 (50.5)	0.661
Within 6 h from symptom onset to CT, *n* (%)	325 (57.3)	223 (59.0)	0.657
Laboratory findings			
WBC	6.64 (5.58–7.89)	6.93 (5.79–8.31)	0.005
HB	130 (124–136)	131 (127–137)	<0.001
PLT	189 ± 25	187 ± 23	0.137
ALT, U/L	46 (41–60)	45 (39–56)	0.098
Albumin, g/dL	38.4 (36.3–41.4)	38.0 (35.9–41.3)	0.231
Cr,	102 (90.2–115)	101 (88.9–113)	0.264
INR	1.10 (0.94–1.28)	1.11 (0.91–1.29)	0.890
HE scores			
PREDICT	4 (2–7)	5 (3–8)	0.017
9 Point	2 (1–3)	2 (1–3)	0.641
BRAIN	7 (3–9)	6 (3–9)	0.051
HE, *n* (%)	135 (23.8)	83 (22.0)	0.560

GCS, Glasgow coma scale; WBC, White blood cell; Hb, Hemoglobin; PLT, Platelet count; ALT, Alanine aminotransferase; Cr, Creatinine; INR, International normalized ratio; HE, Hematoma expansion. † Represented blend sign, hypodensities, black hole sign, swirl sign, island sign, liquification. ‡ Represented admission within 1 h with systolic blood pressure not controlled below 140 mmHg.

### Predictors of HE

The multivariate analysis revealed seven independent predictors of HE: Age, uncontrolled blood pressure, hematoma volume, irregularity/lobulation shape, non-homogeneous density, CT scan performed ≤6 h after symptom onset, and the use of anticoagulation/antiplatelet therapy (Table 2). These variables were chosen based on their established link to HE as reported in existing literature and their availability in routine hospital settings.

**Table 2:** Risk factors for predictive model for HE in the derivation cohort.

Variable	*β* coefficient	Adjusted OR (95%CI)	*P*-value	Point ^a^
Age, y				
<65	Reference			
≥65	1.1266	3.09 (1.77–5.53)	<0.001	1.5
Uncontrolled blood pressure	1.0045	2.73 (1.67–4.52)	<0.001	1.5
Hematoma volume				
≤ 10 ml	Reference			
>10– < 20 ml	2.3654	10.65 (5.33–23.13)	<0.001	3
≥20 ml	2.9458	19.03 (9.12–43.02)	<0.001	4
Irregularity/lobulation shape	1.0110	2.75 (1.68–4.52)	<0.001	1.5
Non-homogeneous density	1.2263	3.41 (2.08–5.76)	<0.001	1.5
Within 6 h from symptom onset to CT	0.9505	2.58 (1.56–4.36)	<0.001	1.5
Anticoagulation/antiplatelet	0.7592	2.14 (1.10–4.15)	0.025	1
Total score				12.5

^a^ Assignment of points to risk factors was based on a linear transformation of the corresponding β regression coefficient. The coefficient of each variable was divided by 0.7592 (the smallest absolute β value, corresponding to Anticoagulation/antiplatelet) and allocated an integer or an half integer score for each variable.

### HE-VSD-A_2_TP score

We developed a novel scoring system specifically designed to predict HE in patients with HICH. We named this scoring system the HE-VSD-A_2_TP score, which is derived from the seven variables it encompasses: Hematoma Volume, Shape, Density, Age, Anticoagulation/antiplatelet, within 6 h from symptom onset to CT, uncontrolled blood Pressure. Each variable was assigned a score proportional to its regression coefficient as determined from the multivariate logistic regression analysis (Table 2). The score for each predictor variable was calculated by dividing the regression coefficient of the variable by the smallest regression coefficient among the significant predictors, ensuring that all scores were on a comparable scale. This process allowed for the transformation of the regression coefficients into clinically interpretable scores. The HE-VSD-A_2_TP score for an individual patient was calculated by summing the scores of each predictor variable that applied to that patient. This cumulative score provided a quantitative estimate of the patient’s risk for HE. The total score ranged from 0 to a maximum of 12.5, with a higher score indicating a greater risk of hematoma expansion.

### Validation of the HE-VSD-A_2_TP score

**Figure 2:**
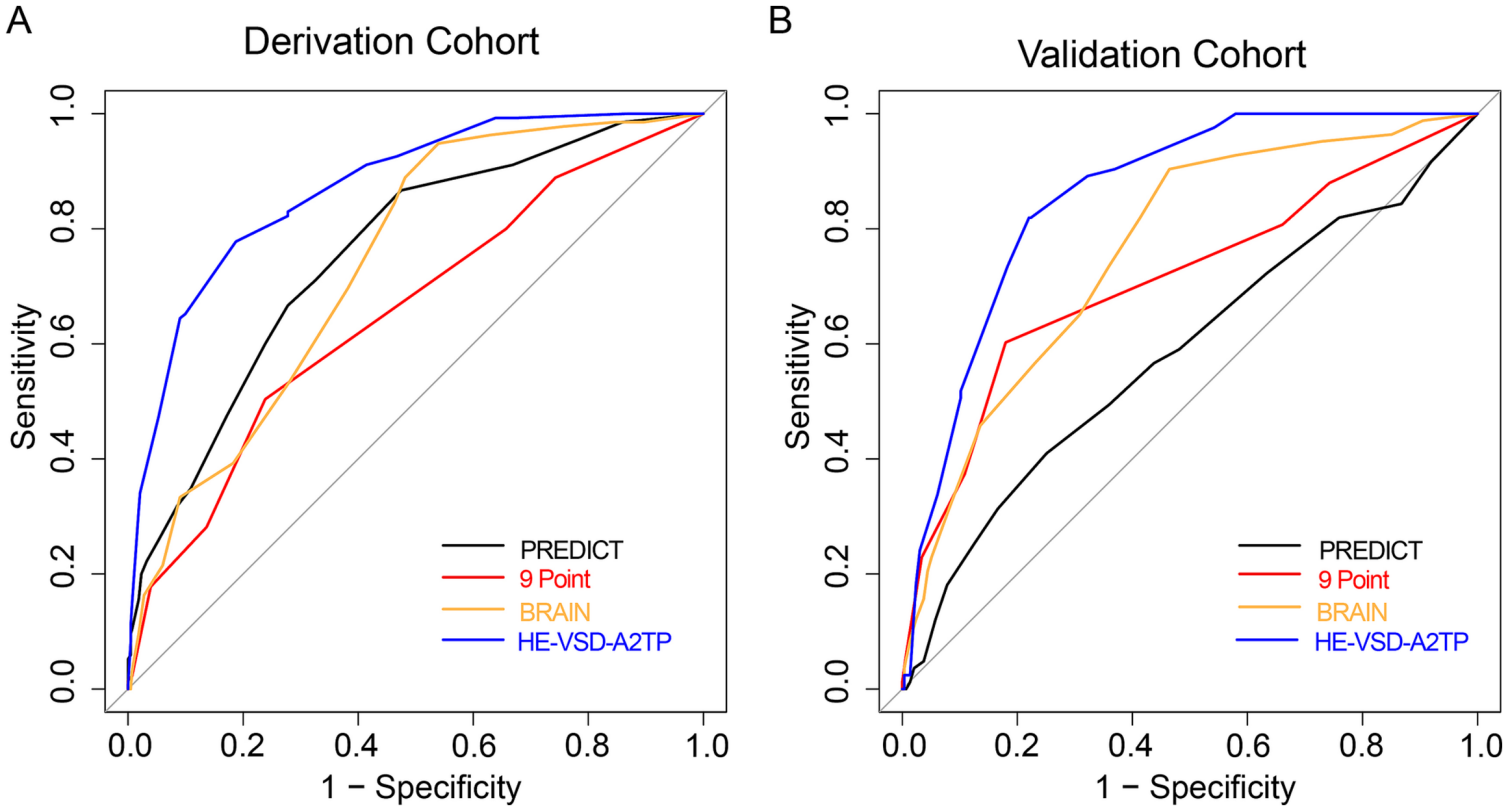
ROC curves for HE prediction in derivation **(A)** and validation **(B)** cohorts.

We compared the performance of the HE-VSD-A_2_TP score with established models, PREDICT, 9 Point and BRAIN score, in predicting HE in patients with HICH. In the derivation cohort, the AUROC for the HE-VSD-A_2_TP score was 0.871 (95% CI, 0.839–0.904), which was significantly higher than that of the PREDICT score at 0.757 (95% CI, 0.711–0.802), the 9 Point score at 0.654 (95% CI, 0.601–0.706), and the BRAIN score at 0.739 (95% CI, 0.695–0.782) (Figure 2A and Table 3). This indicates that the HE-VSD-A_2_TP score demonstrated superior discrimination in predicting HE compared to the other scoring systems. In the validation cohort, the AUROC values for the HE-VSD-A_2_TP score remained high at 0.858 (95% CI, 0.819–0.897) and were significantly higher than those for the PREDICT score at 0.584 (95% CI, 0.510–0.657), the 9 Point score at 0.711 (95% CI, 0.644–0.779), and the BRAIN score at 0.762 (95% CI, 0.707–0.818) (Figure 2B and Table 3). The consistency of the HE-VSD-A_2_TP score’s performance across both cohorts highlights its robustness and generalizability in predicting HE. Furthermore, the HE-VSD-A_2_TP score outperformed the PREDICT, 9 Point, and BRAIN scores in terms of sensitivity, specificity, LHR, NPV, and PPV. The detailed performance metrics for each score are presented in Table 4. The superior performance of the HE-VSD-A_2_TP score across these metrics underscores its clinical utility in more accurately identifying patients at risk of HE.

**Table 3:** Discrimination and calibration of predictive scores to predict HE in derivation and validation cohort.

	Discrimination		Calibration	
	AUROC (95% CI)	*P*-value	H-L Chi-square	*P*-value
Derivation cohort				
PREDICT	0.757 (0.711–0.802)	<0.001	13.855	0.031
9 Point	0.654 (0.601–0.706)	<0.001	11.946	0.008
BRAIN	0.739 (0.695–0.782)	<0.001	33.801	<0.001
HE-VSD-A_2_TP	0.871 (0.839–0.904)		7.569	0.271
Validation cohort				
PREDICT	0.584 (0.510–0.657)	<0.001	8.832	0.357
9 Point	0.711 (0.644–0.779)	<0.001	19.133	<0.001
BRAIN	0.762 (0.707–0.818)	<0.001	14.653	0.066
HE-VSD-A_2_TP	0.858 (0.819–0.897)		6.575	0.254

**Table 4:** Predictive accuracy of scores to predict HE in derivation and validation cohort.

Predictive scores	(95%CI)				LHR (95%CI)	
	Sensitivity	Specificity	PPV	NPV	Positive	Negative
Derivation cohort						
PREDICT	0.867 (0.809–0.924)	0.525 (0.478–0.573)	0.363 (0.311–0.416)	0.927 (0.894–0.959)	1.826 (1.621–2.058)	0.254 (0.164–0.394)
9 Point	0.504 (0.419–0.588)	0.762 (0.721–0.802)	0.398 (0.324–0.471)	0.831 (0.794–0.868)	2.113 (1.666–2.679)	0.652 (0.545–0.779)
BRAIN	0.948 (0.911–0.986)	0.461 (0.414–0.508)	0.355 (0.305–0.404)	0.966 (0.941–0.991)	1.758 (1.598–1.934)	0.113 (0.054–0.233)
HE-VSD-A_2_TP	0.778 (0.708–0.848)	0.812 (0.776–0.849)	0.565 (0.493–0.636)	0.921 (0.894–0.948)	4.148 (3.342–5.148)	0.274 (0.199–0.376)
Validation cohort						
PREDICT	0.410 (0.304–0.515)	0.749 (0.700–0.799)	0.315 (0.227–0.402)	0.819 (0.773–0.864)	1.633 (1.180–2.260)	0.788 (0.651–0.954)
9 Point	0.602 (0.497–0.708)	0.820 (0.777–0.864)	0.485 (0.389–0.582)	0.880 (0.842–0.918)	3.353 (2.484–4.526)	0.485 (0.370–0.635)
BRAIN	0.904 (0.840–0.967)	0.536 (0.479–0.593)	0.354 (0.289–0.418)	0.952 (0.919–0.984)	1.946 (1.689–2.241)	0.180 (0.092–0.351)
HE-VSD-A_2_TP	0.819 (0.736–0.902)	0.780 (0.732–0.827)	0.511 (0.426–0.596)	0.939 (0.909–0.969)	3.718 (2.933–4.714)	0.232 (0.146–0.368)

LHR, likelihood ratio; NPV, negative predictive value; PPV, positive predictive value.

**Figure 3:**
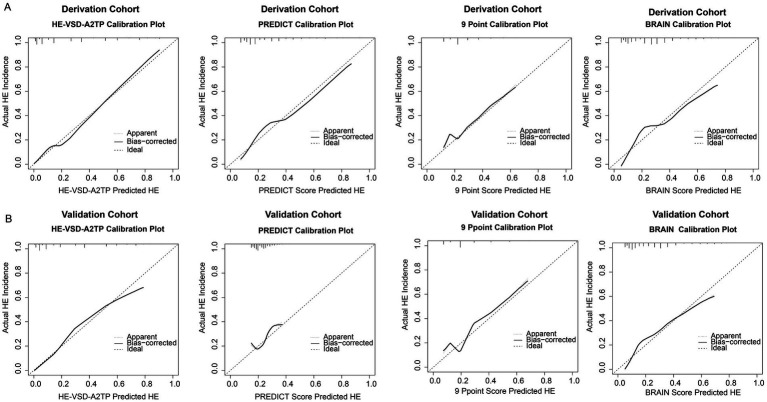
Calibration for HE prediction in derivation **(A)** and validation **(B)** cohorts.

In addition to the discrimination analysis, we also evaluated the calibration of the HE-VSD-A2TP score compared to the PREDICT, 9 Point, and BRAIN scores by H–L test and calibration curves (Figure 3 and Table 3). In the derivation cohort, the HE-VSD-A_2_TP score demonstrated excellent calibration with a H-L Chi-square value of 7.569 and a *p*-value of 0.271. The deviation correction curve generated by the bootstrap method is displayed very close to the reference line, indicating that the HE-VSD-A2TP prediction of HE occurrence is still highly consistent with the actual HE occurrence. In contrast, the PREDICT score, 9 Point score and BRAIN score exhibited the poor calibration (Figure 3A and Table 3). In the validation cohort, the calibration performance of the HE-VSD-A_2_TP score was consistent with that observed in the derivation cohort (Figure 3B and Table 3).

### Risk stratification

The stratification of HE risk in HICH patients was determined using the HE-VSD-A_2_TP score, which was calculated by summing the points assigned to each identified risk factor. The risk stratification was categorized into three groups: low risk (0–4.5 points), moderate risk (5–7 points), and high risk (7.5–12.5 points). The HE-VSD-A_2_TP score effectively categorized patients into low-risk, moderate-risk, and high-risk groups for HE in both the derivation and validation cohorts. Each risk group had a predicted HE rate that closely mirrored the actual HE rates (Table 5).

**Table 5:** Risk of HE in the derivation and validation cohort according to risk stratification.

Risk stratification	*n* (%)	Predicted HE (%)	Actual HE (%)
Derivation cohort			
Low	240 (42.3)	5.17 (4.55–5.20)	4.17
Moderate	141 (24.9)	14.23 (13.73–14.67)	14.18
High	186 (32.8)	56.62 (55.24–58.31)	56.45
Validation cohort			
Low	194 (51.3)	6.09 (2.32–6.21)	4.12
Moderate	69 (18.3)	19.39 (15.35–23.95)	20.29
High	115 (30.4)	52.43 (50.69–54.03)	53.04

The prognostic index was categorized in three groups: low-risk (0–4.5 points), moderate risk (5–7 points), high-risk (7.5–12.5 points).

### Net benefit of using the HE-VSD-A_2_TP score

**Figure 4:**
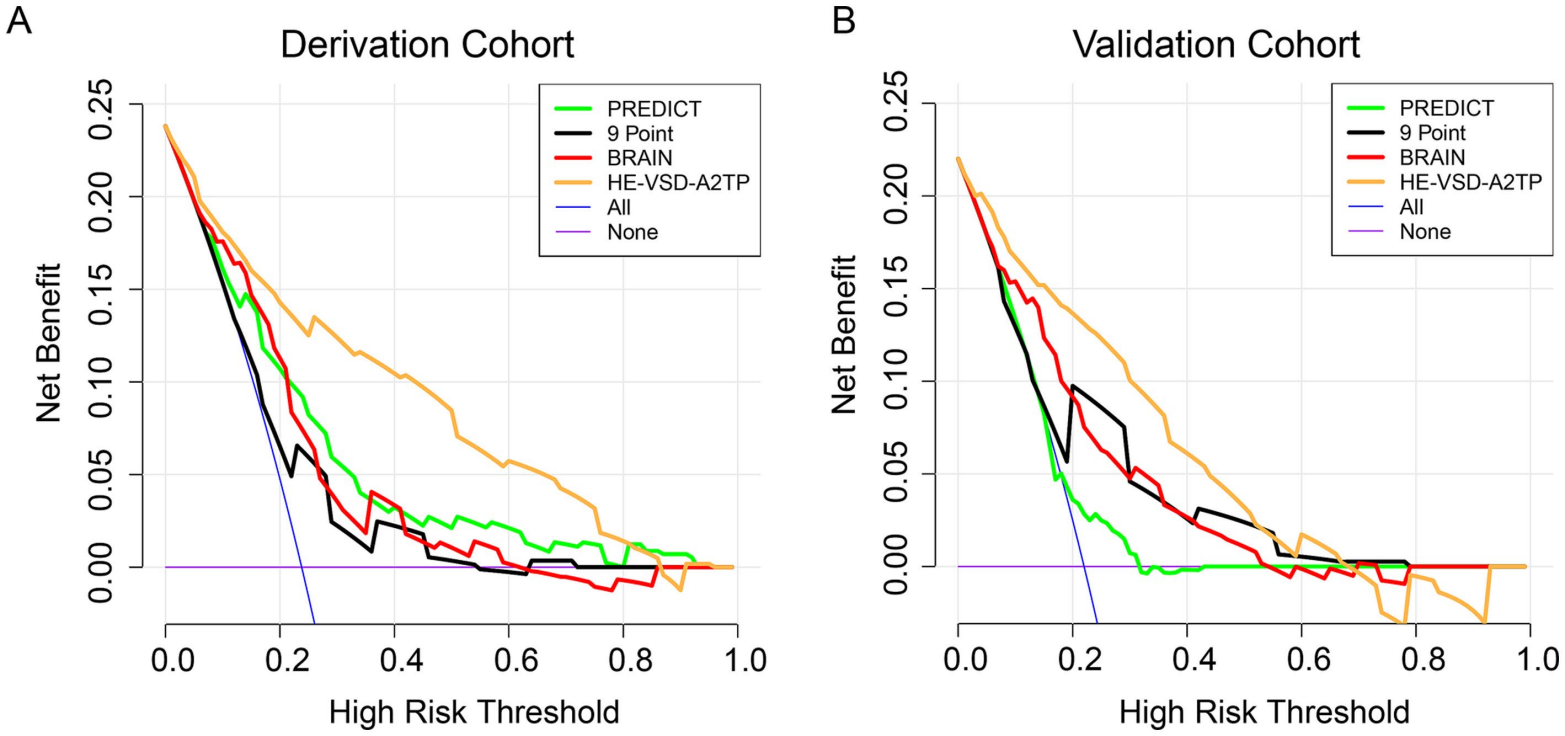
The DCA curve of HE prediction in derivation **(A)** and validation **(B)** cohorts.

Decision curve analysis (DCA) was conducted to evaluate the clinical utility of the HE-VSD-A_2_TP score compared to the PREDICT, 9 Point, and BRAIN scores in both the derivation and validation cohorts (Figure 4). The DCA graphically represents the net benefit of using each scoring system across a range of threshold probabilities for predicting HE. The DCA results from both cohorts consistently show that the HE-VSD-A_2_TP score outperforms the PREDICT, 9 Point, and BRAIN scores in predicting HE. This suggests that the HE-VSD-A_2_TP score is more likely to identify patients who would benefit from targeted interventions to prevent HE, thereby potentially improving patient outcomes.

## Discussion

In this retrospective multicenter study, we developed the HE-VSD-A2TP score to predict HE in ICH patients using routinely available NCCT markers. The score demonstrated higher discriminative accuracy and better calibration than PREDICT, 9-point, and BRAIN scores in our cohorts.

The development of the HE-VSD-A_2_TP score was driven by the need for a robust and widely applicable tool to predict HE in ICH patients. Previous models, while valuable, have shown varying predictive abilities and are not universally applicable (7). For instance, models that rely on CT angiography (CTA) spot sign, such as the PREDICT score and 9-Point score, may not be feasible in all clinical settings due to the unavailability of CTA (9). Moreover, scores focusing on baseline hematoma volume or initial GCS fail to account for the dynamic evolution of ICH and other factors that contribute to HE. Our study addresses these limitations by incorporating a comprehensive set of clinical and imaging features that are readily available in most healthcare settings. The HE-VSD-A_2_TP score is derived from seven independent predictors of HE: hematoma volume, shape, density, age, anticoagulation/antiplatelet use, time from symptom onset to CT, and uncontrolled blood pressure. Each predictor was assigned a score based on its regression coefficient, allowing for a clinically interpretable and easily understood scoring system.

Advanced age has been consistently reported as a predictor of HE, likely due to age-related vascular fragility and decreased brain compliance (12). The significance of hematoma characteristics, such as irregular shape and non-homogeneous density, is supported by studies suggesting these features reflect ongoing hemorrhage and increased risk of expansion (10, 13, 14). Furthermore, the use of anticoagulants and antiplatelet therapy has been implicated in increased risk of HE, presumably due to impaired hemostasis (15). Early blood pressure control, as demonstrated by Ma et al. (16), is crucial in managing ICH, with our study emphasizing the importance of SBP controlled within 1 h after admission. This approach aligns with current clinical guidelines emphasizing the importance of early blood pressure management in ICH (16, 17). Our exclusion of laboratory indicators, which are often subject to delays, reflects our commitment to developing a model based on rapidly obtainable and objective clinical and imaging parameters.

The performance of the HE-VSD-A_2_TP score was favorable compared to existing models like the PREDICT, 9-point, and BRAIN scores in terms of discrimination, calibration, sensitivity, and specificity (18). With an AUC exceeding 0.85 in both cohorts, the HE-VSD-A_2_TP score demonstrates excellent discrimination. This is particularly significant given the heterogeneity in patient populations between the two cohorts, which reflects real-world clinical scenarios. The HE-VSD-A2TP score’s calibration, as assessed by the H-L test and calibration curves, confirms that the model’s predictions are well-aligned with observed outcomes. This is in contrast to the PREDICT, 9-point, and BRAIN scores, which showed poorer calibration in our analysis. Moreover, the score’s sensitivity and specificity highlight its utility in accurately identifying patients at risk of HE.

Risk stratification using the HE-VSD-A_2_TP score allowed for the effective categorization of patients into low-, moderate-, and high-risk groups for HE. The close alignment between predicted and actual risks within each stratum underscores the score’s clinical applicability. This stratification is crucial for guiding clinical decision-making, enabling the prioritization of resources, and potentially improving patient outcomes through targeted interventions (19). For instance, surgery for patients at high risk may be an important treatment to save their lives. DCA further substantiates the clinical utility of the HE-VSD-A_2_TP score, demonstrating its superior net benefit over PREDICT, 9-point, and BRAIN scores across a range of threshold probabilities. This suggests that the HE-VSD-A_2_TP score is more likely to identify patients who would benefit from targeted interventions to prevent HE.

A key strength of the HE-VSD-A_2_TP score is its simplicity, applicability, and the use of objective parameters that can be quickly obtained in diverse clinical settings, as it does not require sophisticated imaging or laboratory tests. It is worth noting that our study has limitations. The retrospective design and the reliance on data from two centers may introduce biases. Additionally, while we endeavored to include a comprehensive set of predictors, unmeasured confounders may exist. We acknowledge the important distinction raised regarding the retrospective nature of our study versus the prospective design of studies like PREDICT. Although our retrospective analysis demonstrated that the HE-VSD-A_2_TP score achieved higher AUC values and better calibration than the PREDICT, 9-point, and BRAIN scores within our cohorts, we agree that the direct comparison of performance metrics across different study designs must be interpreted with caution. The PREDICT score was derived and validated in a prospective setting specifically designed to evaluate the spot sign, which represents a different level of evidence. Our retrospective validation of these existing scores might not fully replicate the conditions of their original prospective studies. The primary contribution of our work lies in proposing a novel score based solely on readily available NCCT and clinical parameters that showed promising predictive ability in our retrospective cohorts. While the observed comparative performance is encouraging, definitive claims of superiority over prospectively derived models would require prospective validation of the HE-VSD-A_2_TP score itself.

In conclusion, the HE-VSD-A_2_TP score represents a significant advancement in the prediction of HE in ICH patients. Its superior predictive performance, ease of use, and applicability in diverse clinical settings make it a promising tool for guiding clinical management strategies and improving patient outcomes.

## Data Availability

The raw data supporting the conclusions of this article will be made available by the authors, without undue reservation.
